# Establishing NIH Community Implementation Programs to improve maternal health

**DOI:** 10.1186/s43058-024-00634-4

**Published:** 2024-09-30

**Authors:** Karen M. Plevock Haase, Candice A. Price, Gina S. Wei, Ilana G. Goldberg, Bryan C. Ampey, Erynn A. Huff, Kimberly R. Durkin, Ashley E. Blair, Camille A. Fabiyi, Keisher S. Highsmith, Melissa S. Wong, David Clark, George A. Mensah

**Affiliations:** 1 Center for Translation Research and Implementation Science, National Heart, Lung, and Blood Institute, National Institutes of Health, Bethesda, Maryland, USA; 2 Division of Cardiovascular Sciences, National Heart, Lung, and Blood Institute, National Institutes of Health, Bethesda, Maryland, USA; 3 Division of Blood Diseases and Resources, National Heart, Lung, and Blood Institute, National Institutes of Health, Bethesda, Maryland, USA; 4 Immediate Office of the Director, National Heart, Lung, and Blood Institute, National Institutes of Health, Bethesda, Maryland, USA; 5 Other Transactions Authority Office, National Heart, Lung, and Blood Institute, National Institutes of Health, Bethesda, Maryland, USA; 6 Office of Management, National Heart, Lung, and Blood Institute, National Institutes of Health, Bethesda, Maryland, USA; 7 Eunice Kennedy Shriver National Institute of Child Health and Human Development, National Institutes of Health, Bethesda, Maryland, USA; 8 Division of Epidemiology, Services and Prevention Research, National Institute On Drug Abuse, National Institutes of Health, Bethesda, Maryland, USA; 9 Department of Obstetrics and Gynecology, Cedars-Sinai Medical Center, Los Angeles, California, USA; 10 Office of Research On Women’s Health, National Institutes of Health, Bethesda, Maryland, USA

**Keywords:** Implementation science, Community engagement, Maternal health, Health disparities

## Abstract

The United States has seen increasing trends of maternal mortality in recent years. Within this health crisis there are large disparities whereby underserved and minoritized populations are bearing a larger burden of maternal morbidity and mortality. While new interventions to improve maternal health are being developed, there are opportunities for greater integration of existing evidence-based interventions into routine practice, especially for underserved populations, including those residing in maternity care deserts. In fact, over 80 percent of maternal deaths are preventable with currently available interventions. To spur equitable implementation of existing interventions, the National Heart, Lung, and Blood Institute launched the Maternal-Health Community Implementation Program (MH-CIP) in 2021. In 2023, the National Institutes of Health’s Implementing a Maternal health and PRegnancy Outcomes Vision for Everyone (IMPROVE) initiative partnered with the NHLBI to launch the IMPROVE Community Implementation Program (IMPROVE-CIP). By design, CIPs engage disproportionately impacted communities and partner with academic researchers to conduct implementation research. This commentary overviews the impetus for creating these programs, program goals, structure, and offers a high-level overview of the research currently supported. Lastly, the potential outcomes of these programs are contextualized within the landscape of maternal health initiatives in the United States.

Contributions to the Literature

- Increase awareness of National Heart, Lung, and Blood Institute’s community implementation science programs focused on maternal health.
- Describe the vision, goals, and research foci for the NIH Community Implementation Programs to improve maternal health equity especially in underserved and minoritized populations.
- Highlight the potential for community-engaged research as an important driver of integrating evidence-based interventions for improved maternal care in communities facing the greatest maternal care disparities and risks for morbidity and mortality.

## Background

The United States is in the midst of a maternal health crisis where 32.9 women die per 100,000 live births as of 2021 [1]. These statistics are even worse depending on various social positions and factors such as race, ethnicity, economic status, and geographic location and age. Of particular concern are maternal health outcomes amongst underserved groups including Black, American Indian and Alaska Native (AI/AN) women. Black women are three times more likely to experience a pregnancy-related complication, when compared to non-Hispanic White women [1]. Compared to non-Hispanic White women, AI/AN individuals are twice as likely to experience severe maternal morbidity and die from pregnancy-related complications [2]. The gap in research addressing maternal health disparities is even greater in these populations around the nation for which much remains incompletely understood in regard to maternal care and outcomes. Moreover, rural residents have a 9% greater risk of severe maternal morbidity and mortality (MMM) in part due to 50% of rural counties having no hospital-based obstetrical services [3], with a greater negative impact on Black and AI/AN individuals. Maternal mortality in women ages 40 and older was 6.8 times more than in women under the age of 25 years [1]. Several additional factors including adverse health conditions (e.g. cardiovascular disease, diabetes), social determinants of health, and social, structural, and systemic factors (e.g., health care systems, access to maternal care, racism and discrimination) all contribute to MMM [1, 4–7].

With over 80% of maternal deaths in the US being preventable [8], there is urgency in scaling up and scaling out existing evidence-based maternal health interventions to improve pregnancy and postpartum outcomes for populations experiencing these disparities. Implementation science prioritizes testing the acceptability, feasibility, affordability, and scalability, for example, of evidence-based interventions to address preventable contributors of poor maternal health outcomes. Implementation science equips practitioners with theories, models, and frameworks to strategically deliver interventions that are both impactful and sustainable [9]. Important research questions that push the field of implementation research in maternal health include: understanding which implementation strategies sustainably improve the implementation of evidence-based maternal health interventions and determining best or promising practices in implementation research to optimize equity by partnering with communities and those with lived-experiences [10].

There is particular promise for equitable community-engaged implementation science to improve health outcomes [11–15]. This has indeed been recognized at the National Institutes of Health (NIH), and over the last several years the NIH has increased focus on community-engaged efforts to improve health in our nation, including major efforts such as the Community Engagement ALliance (CEAL) in 2020 [16]. Also in 2020, the National Heart, Lung, and Blood Institute (NHLBI) launched the Maternal-Health Community Implementation Program (MH-CIP) to focus specifically on maternal health and address equity gaps by developing and strengthening novel community engagement approaches to conducting research. In 2023, the NIH’s Implementing a Maternal health and Pregnancy Outcomes Vision for Everyone (IMPROVE) initiative used this successful community engagement model and partnered with the NHLBI to launch the IMPROVE Community Implementation Program (IMPROVE-CIP).

### Vision and goals

The overall vision of CIP, which includes both MH-CIP and IMPROVE-CIP, is to move towards improved maternal health for all women in the United States and to decrease disparities in maternal health outcomes. The overall goal is to complete community-driven assessments to develop and test strategies used to facilitate the adoption, uptake, scale-up, scale-out, and integration of evidence-based interventions (EBIs) into clinical and community settings. The community driven assessments include a needs assessment (NA) to demonstrate unmet needs in the community, asset mapping (AM) which provides a landscape analysis of existing community resources which can be leveraged to address MMM, and a community priorities assessment (CPA) to align evidence-based interventions with what is most important to the community. Bringing together the needs assessment, asset mapping, and community priorities assessment is critical to CIP success and drives the project designs to fully test identified implementation strategies. Of interest are EBIs proven to improve health outcomes before and during pregnancy and up to one-year post-partum, specifically within disproportionately impacted communities.

### Community and researchers as co-leads is essential

Broad engagement with communities is crucial to understand the impacts and vulnerability of populations most affected by MMM. In particular, community-engaged maternal health research enables and facilitates identification of relevant multi-level factors that affect maternal health within communities. This in turn informs the development of locally driven solutions that are appropriate, acceptable, effective and sustainable in addressing the disproportionate effects of MMM within the engaged communities. As such, a critical component of the CIP platform is the integral partnership and co-leadership between the local academic institutions and embedded community representatives in designing and conducting the research. These community and academic partnered teams are referred to as research coalitions (RCs) where the community representatives and academic researchers work collaboratively as equal partners.

Research coalitions that applied to participate in CIP were required to engage community partners at the onset and throughout the project to successfully lead, partner, advise, and provide feedback to implementation strategy development and testing. This type of involvement required input from the community, partnership in research, and shared leadership. Although community partnerships are established coming into the project, there is the opportunity to add additional partners during the course of the research project. Importantly, study budgets include funds for community partners to be fully engaged and successfully participate in research design and implementation [17]. Operationally, to reflect collaborative efforts and shared leadership, individuals from community partners were required to be identified as Key Personnel including a description of their contributions to the project in their applications. Proposals were to include a broad research team of community engagement researchers and key community members, with at least one Principal Investigator (PI) and/or key personnel from a non-academic institution. Studies that did not include a co-investigator or multiple Principal Investigator (multi-PI) from a community-based organization or community partner were not responsive to this call for applications. Application evaluation criteria included the coalition structure and governance and required letters of commitment from key coalition members. Each RC defined their specific governance structure based on the needs of the community and academic partnership. Prior work has demonstrated that improved health outcomes can be made when there is a concerted and coordinated effort between health care system organizations and communities [18–21]. These community-research partnerships will ensure implementation strategies are feasible and sustainable in the community being served, that EBIs are acceptable and appropriate to the community, and that research questions are aligned with the priorities of that community.

### Spectrum of research projects

The eight RCs funded through the Community Implementation Programs span a broad array of health conditions across the pregnancy spectrum (prepregnancy, pregnancy, postpartum). The maternal health conditions and research foci for these RCs are informed by and guided by community needs and priorities. Some of the leading causes of pregnancy-associated MMM include cardiovascular complications, mental health, and substance use and these are reflected in the RCs’ research. These programs work to mitigate preventable maternal mortality, decrease severe maternal morbidity, and promote health equity. In particular, CIPs work to understand which strategies are most effective at improving uptake and adoption of interventions of known effectiveness while considering local context. Research coalitions consider multiple levels including structural, social, healthcare system, and behavioral factors associated with health disparities to identify and implement sustainable approaches to decrease MMM. Evidence-based practice areas of interest include behavioral interventions such as prevention, early detection, diagnostic, treatment management interventions, and quality improvement programs, with emphasis on culturally and linguistically appropriate strategies. Programs are guided by multidisciplinary, systems, and community-partnered implementation science approaches and utilize existing community-engaged partnerships to reduce MMM related to and associated with pregnancy encompassing the pre-pregnancy, pregnancy and postpartum periods. Examples of studies solicited for CIP included, but were not limited, to (1) testing strategies for implementing effective, evidence-based maternal health care interventions within community settings, and health care models to address structural determinants of health and health disparities of complex patients in diverse systems of care; (2) testing strategies that target organizational structure, climate, culture, and processes to enable dissemination and implementation of clinical/public health information and effective interventions among high-risk populations to improve maternal health equity; (3) testing strategies designed based on community-identified needs, patient reported outcomes (PROs) and patient preference information (PPI) of pre-pregnancy, pregnant, and post-partum individuals; (4) development and testing of dissemination and implementation strategies to improve maternal health and outcomes that are risk-specific for health disparity populations [17]. MH-CIP and IMPROVE-CIP RCs are working with diverse populations including urban, rural, Black, Hispanic, and Indigenous populations, with the goal to improve the health of birthing individuals. The current MH-CIP research foci include promoting preconception care, healthy lifestyle, cardiometabolic health, and appropriate management of severe hypertension. The IMPROVE-CIP research foci include kinship involvement, mental health, substance use disorder, blood pressure management, and prevention of preeclampsia. Collectively, these RCs address high priority areas for maternal health including testing of implementation strategies in maternity care deserts, testing strategies in populations with a high burden of maternal morbidity and mortality, and dissemination of successful strategies to increase uptake and adoption in other similar communities. A high-level overview of these RCs is listed in Table 1.

**Table 1:**
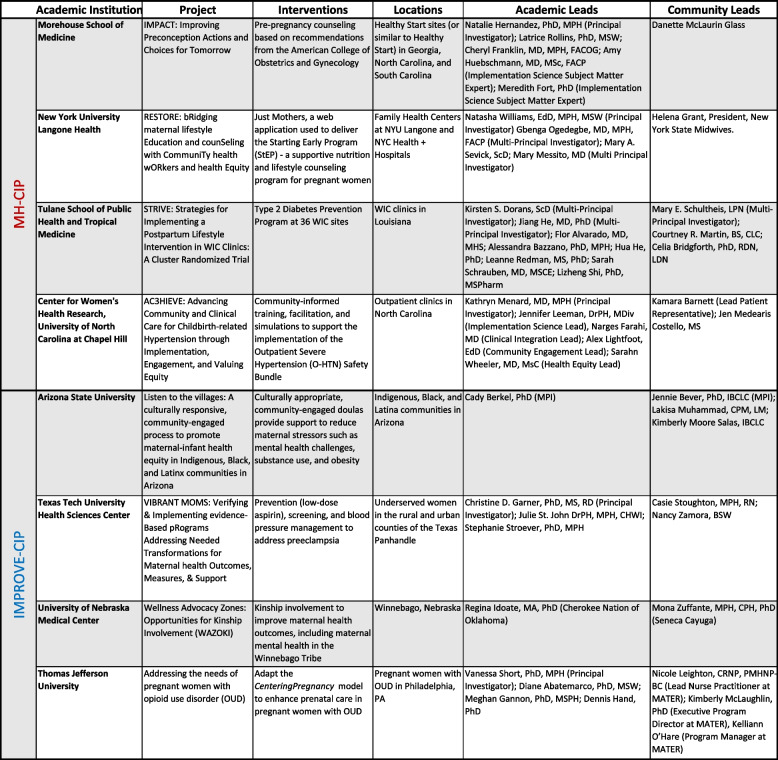
List of MH-CIP RCs that are in Phase II and the IMPROVE-CIP RCs which are early in Phase I. Details in these tables (including community and academic partners) are current as of publication

### Two-phase award

The MH-CIP and IMPROVE-CIP are managed as phased awards with Phase I being a research planning phase and Phase II being a research execution phase. In Phase I, CIP RCs strengthen existing relationships between academic institutions and their community partners. New community organizations may be identified through Phase I assessment activities and these new partnerships are developed and cultivated as needed. Research coalitions complete a community assessment including collecting metrics on community, needs, priorities, and assets, to determine the EBI that will be utilized in Phase II and refine promising implementation strategies to test in Phase II. Community partners serving in a multi-PI role are involved in the design of Phase I and Phase II, including co-leading community needs assessments (e.g. conducting surveys, community member focus groups, asset mapping) and selection of the EBI and strategies to test in Phase II. MH-CIP Phase I lasted approximately 2 years and community and academic partners worked closely over this time period to select the health foci of need and then appropriately tailored the EBI and implementation strategies to ensure the health care solutions fit the community health needs and priorities. IMPROVE-CIP is currently undergoing a two-year Phase I where RCs are likewise completing community assessments and tailoring of EBIs and implementation strategies to promote acceptability, usability, adoption, and sustainment of EBIs within their communities.

Research Coalitions are required to submit a transition request package to move from the research planning phase (Phase I) to the research implementation phase (Phase II). This transition request package includes a (1) community engagement plan, (2) the report of the community needs assessment, asset mapping, and the community priorities assessment, (3) letters of support from community partners, and (4) the phase II protocol synopsis that must include how Phase I findings with the community impacted their Phase II design. These materials go through a rigorous external and internal review with a key emphasis on how community were truly leading the research design. When RCs enter Phase II, they continue to work closely with their communities to collect metrics to demonstrate impact and outcomes throughout the Phase II project period of performance. They also assess the potential for sustainability in community-settings beyond the project time frame. Sustainability, in this context, could include plans for long-term integration of the EBI into community settings after the study period and any necessary financial, organizational, and personnel supports. Research coalitions are laying a strong foundation for potential expansion into other community-settings and geographic areas.

An important element of MH-CIP and IMPROVE-CIP is that the RCs are funded using an Other Transactions (OT) agreement. The Other Transactions Authority (OTA) gives increased flexibility for federal agencies to partner with traditional and non-traditional research and community organizations/entities. The OTA allows for partners to include those that the government does not typically engage including community-based organizations (CBOs). However, due to the infrastructure needed to manage the award and disperse funds it would likely be overly burdensome for CBOs. Thus in the case of CIPs the award is to the academic institution. The academic institution serves as the fiscal agent as there are typically established resources and business officials to manage the federal funds. To combat fiscal inequities that may be present in projects that work with community partners, CIPs, which are under the Community Engagement ALliance (CEAL) follows CEAL best practices to require at least 25% of funds going to community partners. The OTA allows for flexibility, including the ability to adjust agreements in an agile environment while remaining responsive to agency priorities unlike traditional grants and contracts. This mechanism is used when traditional grants or contracts would not be appropriate. The OT agreement provides the MH-CIP and IMPROVE-CIP programs the ability to collaborate and partner with community organizations and the private sector and promotes trust in the spirit of cooperation with priority populations. The flexibility created by the use of an OTA aids in RCs to progress at the speed of the community trust, particularly during Phase I as RCs design their implementation strategies per community input. This supports RCs’ ability to foster and maintain trust in their respective communities. As such, NIH program staff work closely with the awarded RCs and bilateral agreement is needed as projects are designed, especially as RCs are moving from Phase I to Phase II. This allows for the scope to be modified quickly while capturing crucial community inputs.

By the end of the program, RCs will have tested promising implementation strategies in close partnership between community and academic researchers, and there will be a clear understanding of what does (or does not) work to implement the EBI in the specified community setting(s). Additionally, new knowledge of effective strategies for implementation of maternal health interventions will be disseminated and allow for widespread adoption and integration of EBIs into critical maternal care settings. Inclusion of historically under-represented communities will help reduce knowledge gaps about how to equitably disseminate EBIs in community settings. Importantly, lessons learned, and knowledge gained about successful implementation strategies will move us towards improved maternal health in our nation (and beyond).

### Community Engagement Technical Assistance Center coordinates across programs

The Community Engagement Technical Assistance Center (CETAC) serves as the prime awardee for the CIP programs. As the prime awardee, CETAC manages scientific and administrative oversight of the programs including the cross-program evaluation that will include measures of community engagement. Research coalitions submit monthly and annual reports and meet monthly with CETAC to report progress toward project milestones. As part of these reports and meetings, CETAC collects extensive data regarding the level of involvement of each community partner, which may range from providing input (advise) to true shared leadership (govern). Additionally, the CETAC team is collecting data across the RCs using an implementation strategy log. This log is modeled after the Longitudinal Implementation Strategy Tracking System (LISTS) tool [22]. Although RCs were not required to use a specific implementation framework, this log will match implementation strategies to common implementation frameworks to systematically document and track the use and adaptation of strategies as RCs implement their interventions. Although we do not report out fully on the operationalization of this implementation strategy log here, this is a key feature for cross analysis of the program. CETAC also facilitates a monthly Learning Community meeting that brings together all community and academic partners in a collaborative space for sharing best practices, discussing challenges and solutions, and encouraging innovation to strengthen community partnerships. A final evaluation of the CIP programs will evaluate community engagement across the award based on data collected on a monthly basis through milestone monitoring, annual reports from RCs, and the implementation science logs that are updated as RCs move through their research implementation phase.

## Conclusion and long-term vision

Through programs like MH-CIP and IMPROVE-CIP, RCs build on existing and new relationships between local academic institutions and embedded community organizations to navigate the challenges and opportunities for successfully implementing maternal health interventions in underserved communities. Indeed, the promise of implementation science to close gaps in maternal care has also recently been highlighted by researchers utilizing a modified Delphi method to gather and rank implementation research priorities to address the maternal health crisis in the United States [10]. Overall, through these initiatives and growing momentum in the field, it is envisioned that the U.S. will move towards narrowing the maternal health equity gap and improve maternal health outcomes across multiple communities and settings.

## Data Availability

Not applicable.

## Abbreviations

AI/AN: American Indian/Alaska Native
AM: Asset Mapping
CEAL: Community Engagement ALliance
CIP: Community Implementation Program
CPA: Community Priorities Assessment
EBIs: Evidence-Based Interventions
IMPROVE: Implementing a Maternal health and PRegnancy Outcomes Vision for Everyone
IMPROVE-CIP: Implementing a Maternal health and PRegnancy Outcomes Vision for Everyone Community Implementation Program
MH-CIP: Maternal Health Community Implementation Program
MMM: Maternal morbidity and mortality
NA: Needs Assessment
NHLBI: National Heart, Lung, and Blood Institute
OT: Other Transactions
OTA: Other Transactions Authority
RC: Research Coalition
